# Electronic Properties of Linear and Cyclic Boron Nanoribbons from Thermally-Assisted-Occupation Density Functional Theory

**DOI:** 10.1038/s41598-019-48560-z

**Published:** 2019-08-20

**Authors:** Sonai Seenithurai, Jeng-Da Chai

**Affiliations:** 1 Department of Physics, National Taiwan University, Taipei, 10617 Taiwan; 2 Center for Theoretical Physics and Center for Quantum Science and Engineering, National Taiwan University, Taipei, 10617 Taiwan

**Keywords:** Theoretical chemistry, Computational chemistry, Density functional theory, Quantum chemistry, Chemical physics, Chemistry, Physics, Nanoscience and technology, Nanoscale materials, Electronic properties and materials

## Abstract

It remains rather difficult for traditional computational methods to reliably predict the properties of nanosystems, especially for those possessing pronounced radical character. Accordingly, in this work, we adopt the recently formulated thermally-assisted-occupation density functional theory (TAO-DFT) to study two-atom-wide linear boron nanoribbons *l*-BNR[2,*n*] and two-atom-wide cyclic boron nanoribbons *c*-BNR[2,*n*], which exhibit polyradical character when the *n* value (i.e., the number of boron atoms along the length of *l*-BNR[2,*n*] or the circumference of *c*-BNR[2,*n*]) is considerably large. We calculate various electronic properties associated with *l*-BNR[2,*n*] and *c*-BNR[2,*n*], with *n* ranging from 6 to 100. Our results show that *l*-BNR[2,*n*] and *c*-BNR[2,*n*] have singlet ground states for all the *n* values examined. The electronic properties of *c*-BNR[2,*n*] exhibit more pronounced oscillatory patterns than those of *l*-BNR[2,*n*] when *n* is small, and converge to the respective properties of *l*-BNR[2,*n*] when *n* is sufficiently large. The larger the *n* values, the stronger the static correlation effects that originate from the polyradical nature of these ribbons. Besides, the active orbitals are found to be delocalized along the length of *l*-BNR[2,*n*] or the *c*ircumference of *c*-BNR[2,*n*]. The analysis of the size-dependent electronic properties indicates that *l*-BNR[2,*n*] and *c*-BNR[2,*n*] can be promising for nanoelectronic devices.

## Introduction

Boron is as versatile as carbon in forming different nanostructures. Over the past few decades, boron nanomaterials have been explored extensively, and there has been growing interest in the investigation of boron nanomaterials, due to their interesting properties and potential applications in electronics and other industries^1–8^. The recent interest in boron and other nanomaterials has been partly inspired by the structures, properties, and applications of carbon nanomaterials (e.g., the C_60_ fullerene, carbon nanotubes (CNTs), and graphene)^9–11^. The prediction of B_80_ fullerene^12^, observation of B_40_ fullerene^13^, and observation of the Dirac cone in borophene^9,14,15^ have also shown promise in electronics and industrial applications. Besides, boron nanomaterials have also been proposed for supercapacitor^16^, Li-ion battery^17,18^, molecular Wankel motor^6^, and hydrogen storage^19,20^ applications.

The vast boron nanomaterials can be planar or quasi-planar with single or multiple hexagonal vacancies, tubular, etc. The clusters can be neutral, cationic, or anionic. Among various boron nanomaterials, one-dimensional (1D) and quasi-1D nanostructures^4,5,8,21–24^ are of great interest for nanoelectronics applications. In the case of 1D boron nanostructures, the unique properties of CNTs have motivated interest in boron nanotubes^4^. For CNTs, the chirality-dependent electronic properties are interesting, but it remains challenging to synthesize CNTs with uniform chirality, which is necessary for electronics applications. Thus, novel 1D structures based on boron have also been intensively studied in recent years. With the theoretical predictions of boron fullerenes and 2D sheets, obtaining the possible structures and properties of 1D and quasi-1D boron nanomaterials can be the obvious next step.

Very recently, Liu *et al*.^24^ studied the mechanochemical properties of 1D boron chains (also known as linear boron chains) and quasi-1D boron nanoribbons (also known as two-atom-wide linear boron nanoribbons (which can be the narrowest linear boron nanoribbons)). The study revealed that the quasi-1D boron nanoribbons are more stable than the 1D boron chains. These boron chains and nanoribbons exhibit attractive mechanochemical property (i.e., stress-dependent structural transition between the quasi-1D boron nanoribbons and 1D boron chains). The quasi-1D boron nanoribbons are metallic in equilibrium. However, when they are stretched, the quasi-1D boron nanoribbons can morph into the antiferromagnetic semiconducting 1D boron chains. On the other hand, when the stretched 1D boron chains are released, they can fold back into the metallic quasi-1D boron nanoribbons. The 1D boron chains and quasi-1D boron nanoribbons have very high mechanical stiffness of 46 to 72 eV/Å. These interesting mechanochemical properties make these boron chains and boron nanoribbons potential materials for constant-force springs at the nanoscale^24^.

**Figure 1:**
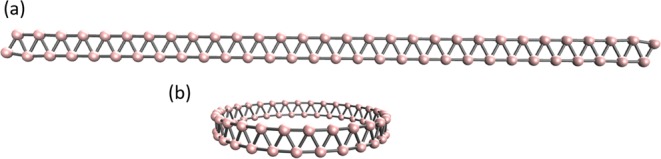
Structures of (**a**) *l*-BNR[2,30] and (**b**) *c*-BNR[2,30], each containing 30 boron atoms along the length or circumference of ribbon.

As illustrated in Fig. 1(a), a two-atom-wide linear boron nanoribbon with *n* boron atoms along the length of ribbon, which is designated as *l*-BNR[2,*n*], is studied in the present work. Besides, its cyclic isomer, a two-atom-wide cyclic boron nanoribbon with *n* boron atoms along the circumference of ribbon (see Fig. 1(b)), which is denoted as *c*-BNR[2,*n*], is also studied here. Note that *c*-BNR[2,*n*] may find applications in molecular motors, nanoscale devices, electronics, etc. In addition, *c*-BNR[2,*n*] can be regarded as the building blocks of boron nanotubes. It has been reported that boron *α*-sheets are metallic. However, when they are wrapped to form single-walled boron nanotubes, the latter (with a diameter less than 20 Å) become semiconducting. The metal-to-semiconductor transition is due to the curvature-induced surface buckling^25^. The experimentally observed boron nanowires with diameters ranging from 20 to 200 nm and lengths up to a few *μ*m have been found to be semiconducting in nature^26^. Therefore, a comprehensive study on the electronic properties of *l*-BNR[2,*n*] and *c*-BNR[2,*n*] may provide further insight into the development and applications of boron nanotubes as well.

As of now, the studies of *l*-BNR[2,*n*]^24,27,28^ and *c*-BNR[2,*n*]^29–31^ remain very scarce. Recent computational studies have shown that B_*n*_${{\rm{H}}}_{2}^{2-}$ (up to *n* = 22)^32^, Li_2_B_*n*_H_2_ (up to *n* = 22)^32^, and B_12_${{\rm{F}}}_{n}^{0/-}$ (*n* = 1–6)^33^ have structures similar to *l*-BNR[2,*n*]. Experimentally, a photoemission spectroscopy study has revealed the presence of dihydrogenated boron clusters H_2_${{\rm{B}}}_{n}^{-}$ (*n* = 7–12), which also have structures similar to *l*-BNR[2,*n*]^27^. Besides, crystalline boron nanoribbons with widths ranging from 20 to 100 nm have been successfully synthesized and characterized by Xu *et al*.^3^. However, it remains very difficult to synthesize *l*-BNR[2,*n*]. On the other hand, while single-walled boron nanotubes have been successfully synthesized^34,35^, the syntheses of *c*-BNR[2,*n*] have not been realized yet. The difficulty in synthesizing *l*-BNR[2,*n*] and *c*-BNR[2,*n*] may be due to the presence of strong static correlation effects in these materials (commonly occurring in low-dimensional structures because of the effect of quantum confinement^36^). Therefore, a computational study on the electronic properties of *l*-BNR[2,*n*] and *c*-BNR[2,*n*] with various *n* values, can be essential for the progress in this field, and may also play an important role in the selection of ideal materials for nanoelectronics applications.

Among various computational methods, presently, Kohn-Sham density functional theory (KS-DFT)^37^ remains very popular, because of the desired balance between accuracy and efficiency. Nonetheless, KS-DFT with approximate exchange-correlation (XC) density functionals can have spectacular failures (e.g., the self-interaction error, static correlation error, etc.) in certain situations^38,39^. In particular, KS-DFT with conventional semilocal, hybrid, and double-hybrid XC density functionals cannot adequately describe the ground-state properties of systems with radical nature, such as the larger *l*-BNR[2,*n*]/*c*-BNR[2,*n*] (as will be shown later). Typically, accurate multi-reference (MR) computational approaches are required for the study of systems with radical nature^40–45^. Nevertheless, due to the expensive computational cost, calculations based on accurate MR computational approaches are applicable only for small systems, and become intractable for large systems (especially for geometry relaxation). Accordingly, nanosystems with radical nature are beyond the reach of traditional computational methods. Since the number of electrons in *l*-BNR[2,*n*]/*c*-BNR[2,*n*] quickly increases with the system size (*n*), calculations based on the presently available MR computational approaches are unlikely to be feasible, especially for the larger *l*-BNR[2,*n*]/*c*-BNR[2,*n*].

Aiming to resolve the aforementioned problem, we have developed TAO-DFT (thermally-assisted-occupation density functional theory)^46^ for the study of nanosystems with radical nature in recent years. Note that TAO-DFT, which employs fractional orbital occupations generated by the Fermi-Dirac distribution (governed by a fictitious temperature *θ*), reduces to KS-DFT in the absence of strong static correlation, allowing a well-balanced description for both systems with non-radical nature and systems with radical nature. Within the framework of TAO-DFT, the presently available local density approximation (LDA)^46^, generalized-gradient approximation (GGA)^47^, and global hybrid^48^ XC density functionals can also be adopted. Besides, we have also developed a scheme for the self-consistent determination of *θ* to improve the overall accuracy of TAO-DFT for general applications^49^. To demonstrate its applicability, we have recently employed TAO-DFT for the study of the electronic properties of several nanosystems with radical nature, including acenes^46–48^, zigzag graphene nanoribbons^50^, cyclacenes^51^, Möbius cyclacenes^52^, alternant polycyclic aromatic hydrocarbons^53^, and the coronene series^54^. Besides, we have also employed TAO-DFT to search for desirable hydrogen storage materials among nanosystems with radical nature in recent years^19,55,56^. Very recently, TAO-DFT and related methods have also been successfully applied to study the electronic properties of cyclic nanorings and single-walled CNTs by other research groups^57,58^. Accordingly, in this work, we employ TAO-DFT to study the electronic properties of *l*-BNR[2,*n*] and *c*-BNR[2,*n*], with *n* ranging from 6 to 100.

## Computational Details

We perform all calculations with Q-Chem 4.4^59^, using the 6–31 G(d) basis set and the numerical grid containing 75 Euler-Maclaurin radial grid points and 302 Lebedev angular grid points. Results are obtained from TAO-LDA^46^ (i.e., TAO-DFT with the LDA exchange, correlation, and *θ*-dependent density functionals) with the fictitious temperature *θ* = 7 mhartree.

While more complicated XC functionals (e.g., the GGA^47^ and global hybrid^48^ XC functionals) may be employed in TAO-DFT as well, they outperform TAO-LDA primarily for the properties closely related to short-range XC effects (e.g., the atomization energies and barrier heights of systems with non-radical nature), not for the properties closely related to static correlation (e.g., the singlet-triplet energy gaps and fundamental gaps of systems with radical nature)^46–48^. For example, the GGA and global hybrid XC functionals in TAO-DFT were found to perform similarly to TAO-LDA for the electronic properties of linear acenes (i.e., systems with polyradical nature)^46–48^. Consequently, the electronic properties of *l*-BNR[2,*n*] and *c*-BNR[2,*n*] from TAO-LDA should be qualitatively similar to those from the GGA and global hybrid XC functionals in TAO-DFT.

It is worth mentioning that for TAO-LDA, the vertical ionization potential, vertical electron affinity, and fundamental gap of a molecule cannot be directly calculated using the negative of the highest occupied molecular orbital (HOMO) energy, the negative of the lowest unoccupied molecular orbital (LUMO) energy, and the HOMO-LUMO gap (i.e., the energy difference between the HOMO and LUMO), respectively, due to the possibility of fractional orbital occupations (see, e.g., Section III of ref.^49^). Therefore, in this work, the vertical ionization potential, vertical electron affinity, and fundamental gap of *l*-BNR[2,*n*]/*c*-BNR[2,*n*] from TAO-LDA are obtained with multiple energy-difference calculations (see Equations (2) to (4)).

## Results and Discussion

### Singlet-triplet energy gap

In order to determine the ground state of *l*-BNR[2,*n*]/*c*-BNR[2,*n*] (*n* = 6–100), we perform calculations based on spin-unrestricted TAO-LDA to obtain the lowest singlet and lowest triplet states of *l*-BNR[2,*n*]/*c*-BNR[2,*n*], with the respective structures being fully optimized. Subsequently, we calculate the singlet-triplet energy gap (i.e., ST gap)^19,46–48,50–52,55,56^ of *l*-BNR[2,*n*]/*c*-BNR[2,*n*] as

1 $${E}_{{\rm{ST}}}={E}_{{\rm{T}}}-{E}_{{\rm{S}}}.$$

Here, *E*_S_ and *E*_T_ are the lowest singlet and lowest triplet energies, respectively, of *l*-BNR[2,*n*]/*c*-BNR[2,*n*].

**Figure 2:**
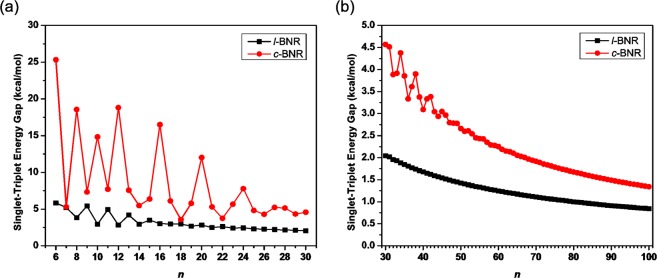
Singlet-triplet energy gap of *l*-BNR[2,*n*]/*c*-BNR[2,*n*] ((**a**) *n* = 6–30 and (**b**) *n* = 30–100), obtained from spin-unrestricted TAO-LDA.

As shown in Fig. 2, *E*_ST_ decreases with an oscillatory pattern as *n* increases. Nonetheless, the oscillations are damped, and eventually vanish with the increase of *n*. The *E*_ST_ value of *c*-BNR[2,*n*] exhibits a more pronounced oscillatory pattern than that of *l*-BNR[2,*n*] when *n* is small, and monotonically converges from above to the *E*_ST_ value of *l*-BNR[2,*n*] when *n* is sufficiently large. Besides, for considerably large *n* (e.g., *n* > 30 for *l*-BNR[2,*n*] or *n* > 60 for *c*-BNR[2,*n*]), the *E*_ST_ values of *l*-BNR[2,*n*] and *c*-BNR[2,*n*] monotonically decrease with the increase of molecular size. Note also that *c*-BNR[2,*n*] possesses a larger *E*_ST_ value than *l*-BNR[2,*n*]. On the basis of our TAO-LDA results, for all the systems investigated (*n* = 6–100), *l*-BNR[2,*n*] and *c*-BNR[2,*n*] have singlet ground states (see Table S1 in Supplementary Information). However, the reason for the oscillations appeared on the smaller *l*-BNR[2,*n*] and *c*-BNR[2,*n*] may not be obvious, and hence, it will be interesting to build a simple model to explain this fact in the near future.

Note that the lowest singlet energies of *l*-BNR[2,*n*]/*c*-BNR[2,*n*] obtained with spin-restricted and spin-unrestricted calculations should be the same for the exact theory due to the symmetry constraint^46–48,60^. Nonetheless, KS-DFT with conventional XC functionals may not satisfy this condition, especially for systems with radical nature^41,42,46–51,60^. Here, we examine if the symmetry-breaking effects occur by additionally performing spin-restricted TAO-LDA calculations for the lowest singlet states of *l*-BNR[2,*n*] and *c*-BNR[2,*n*], with the respective structures being completely optimized. The difference between the spin-restricted and spin-unrestricted energies, obtained with TAO-LDA, for the lowest singlet state of *l*-BNR[2,*n*]/*c*-BNR[2,*n*] is essentially zero (i.e., within the numerical precision considered in the present work), showing that unphysical symmetry-breaking solutions are not generated by our spin-unrestricted TAO-LDA calculations.

Note that the singlet-triplet energy gaps of molecules are essential to understand many chemical processes. For example, there has recently been great interest in incorporating the singlet-fission phenomenon in solar energy conversion due to the improved energy conversion efficiency. As the singlet-triplet energy gaps and the energetics of the singlet fission are closely related, accurate prediction of the singlet-triplet energy gaps of molecules is critically important^61–64^. Besides, molecules with small singlet-triplet energy gaps are expected to be useful for thermally activated delayed fluorescence (TADF) applications^63,65^. Therefore, the singlet-triplet energy gaps of *l*-BNR[2,*n*] and *c*-BNR[2,*n*] reported in this work may provide insight into the singlet-fission phenomenon and TADF applications, which can be helpful for solar energy applications.

### Vertical ionization potential, vertical electron affinity, and fundamental gap

It is interesting to examine whether *l*-BNR[2,*n*] and *c*-BNR[2,*n*] are useful for photovoltaic applications. Spin-unrestricted TAO-LDA calculations are carried out, at the ground-state structure of *l*-BNR[2,*n*]/*c*-BNR[2,*n*], to determine the vertical ionization potential^19,47,48,50–52,55,56^.

2 $${{\rm{IP}}}_{v}={E}_{tot}({\rm{cation}})-{E}_{tot}({\rm{neutral}}),$$

vertical electron affinity

3 $${{\rm{EA}}}_{v}={E}_{tot}({\rm{neutral}})-{E}_{tot}({\rm{anion}}),$$

and fundamental gap

4 $${E}_{g}={{\rm{IP}}}_{v}-{{\rm{EA}}}_{v}.$$

Here, *E*_*tot*_(neutral), *E*_*tot*_(cation), and *E*_*tot*_(anion) are the total energies of *l*-BNR[2,*n*]/*c*-BNR[2,*n*] in the neutral, cationic, and anionic states, respectively.

**Figure 3:**
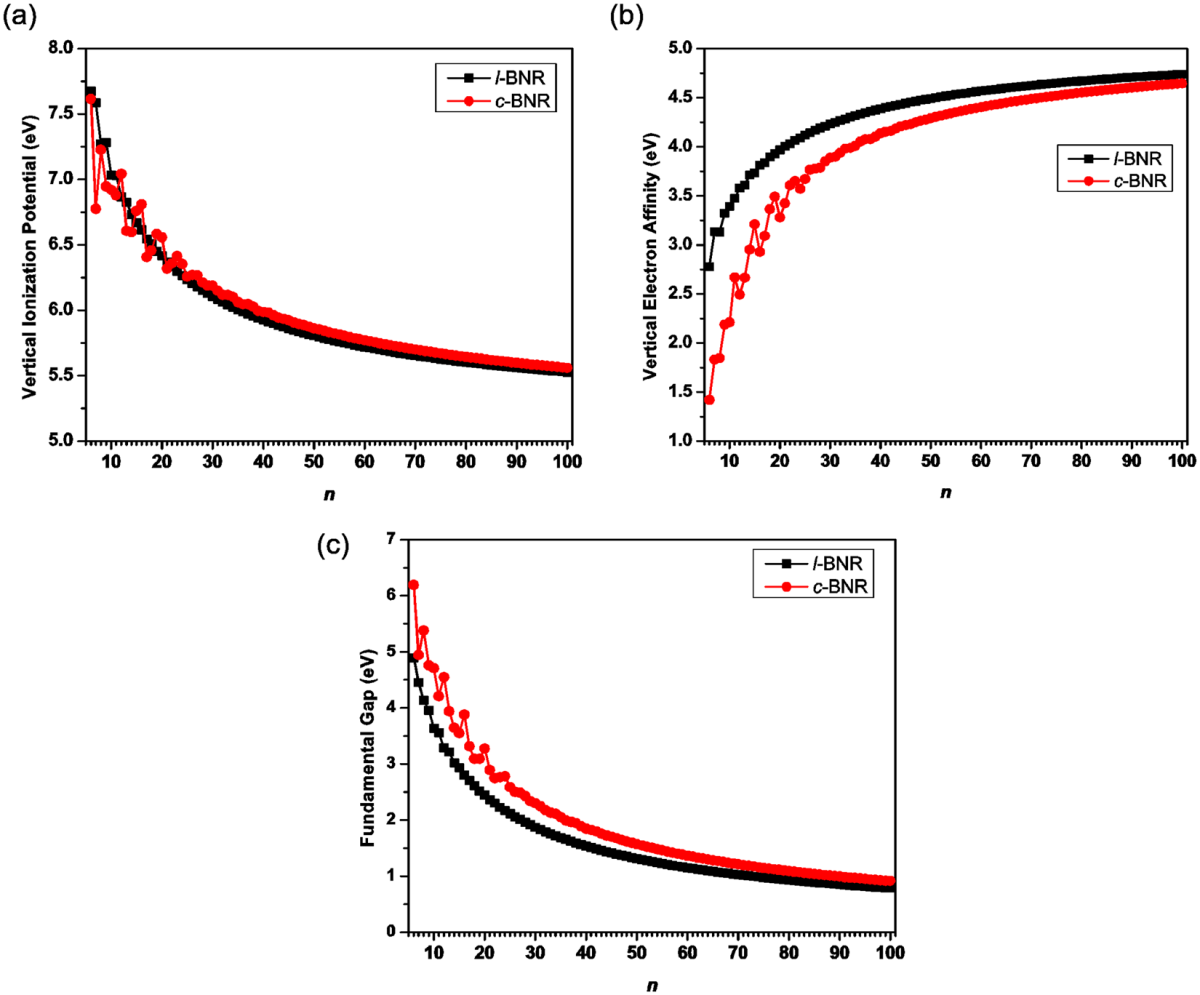
(**a**) Vertical ionization potential, (**b**) vertical electron affinity, and (**c**) fundamental gap for the ground state of *l*-BNR[2,*n*]/*c*-BNR[2,*n*] (*n* = 6–100), obtained from spin-unrestricted TAO-LDA.

As the system size increases, IP_*v*_ (see Fig. 3(a)) generally monotonically decreases (with a slight oscillatory pattern only for the smaller *l*-BNR[2,*n*] (*n* ≤ 10) or smaller *c*-BNR[2,*n*] (*n* ≤ 30)), EA_*v*_ (see Fig. 3(b)) generally monotonically increases (with a slight oscillatory pattern only for the smaller *l*-BNR[2,*n*] (*n* ≤ 10) or smaller *c*-BNR[2,*n*] (*n* ≤ 30)), and *E*_*g*_ (see Fig. 3(c)) generally monotonically decreases (with a slight oscillatory pattern only for the smaller *c*-BNR[2,*n*] (*n* ≤ 30)).

Note that *c*-BNR[2,*n*] possesses a larger *E*_*g*_ value than *l*-BNR[2,*n*]. Besides, the *E*_*g*_ values of *l*-BNR[2,*n*] (*n* = 15–73) and *c*-BNR[2,*n*] (*n* = 21–89) range from 1 eV to 3 eV, lying in the ideal region relevant to solar energy applications. Our theoretical results for IP_*v*_, EA_*v*_, and *E*_*g*_ (see Tables S2 and S3 in Supplementary Information) may guide further experimental studies on *l*-BNR[2,*n*] and *c*-BNR[2,*n*].

### Symmetrized von Neumann entropy

In view of the smaller *E*_ST_ and *E*_*g*_ values, the larger *l*-BNR[2,*n*]/*c*-BNR[2,*n*] are expected to possess more pronounced radical character in their ground states than the shorter *l*-BNR[2,*n*]/*c*-BNR[2,*n*]. To provide a quantitative measure of the radical character of *l*-BNR[2,*n*]/*c*-BNR[2,*n*], spin-unrestricted TAO-LDA calculations are carried out, at the ground-state structure of *l*-BNR[2,*n*]/*c*-BNR[2,*n*], to obtain the symmetrized von Neumann entropy^19,47,48,50–52,55,56,60^.

5 $${S}_{{\rm{v}}{\rm{N}}}=-\,\frac{1}{2}\sum _{\sigma =\alpha ,\beta }\,\mathop{\sum }\limits_{i=1}^{{\rm{\infty }}}\{{f}_{i,\sigma }{\rm{l}}{\rm{n}}({f}_{i,\sigma })+(1-{f}_{i,\sigma }){\rm{l}}{\rm{n}}(1-{f}_{i,\sigma })\}.$$

Here, *f*_*i*,*σ*_ (i.e., a value between 0 and 1) is the *i*^th^ *σ*-spin (i.e., *α*-spin or *β*-spin) orbital occupation number obtained with spin-unrestricted TAO-LDA, approximately yielding the *i*^th^ *σ*-spin natural orbital occupation number^46–48,53^. For a system with non-radical nature ({*f*_*i*,*σ*_} take values in the vicinity of 0 or 1), *S*_vN_ is rather small. However, for a system with radical nature ({*f*_*i*,*σ*_} can differ greatly from either 0 or 1 for spin-orbitals with noticeable fractional occupations (i.e., active spin-orbitals), and take values in the vicinity of 0 or 1 for other spin-orbitals), the corresponding *S*_vN_ can grow rapidly with the number of spin-orbitals that possess noticeable fractional occupations.

**Figure 4:**
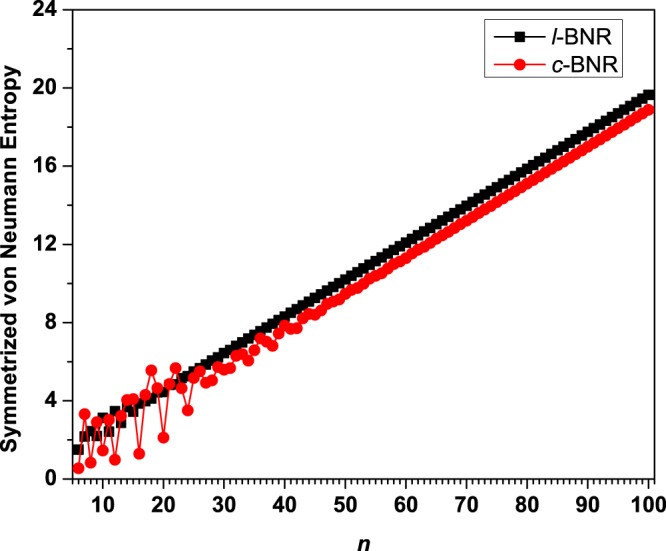
Symmetrized von Neumann entropy for the ground state of *l*-BNR[2,*n*]/*c*-BNR[2,*n*] (*n* = 6–100), obtained from spin-unrestricted TAO-LDA.

With the increase of system size, *S*_vN_ (see Fig. 4) generally monotonically increases (with a slight oscillatory pattern only for the smaller *l*-BNR[2,*n*] (*n* ≤ 20) or smaller *c*-BNR[2,*n*] (*n* ≤ 50)), implying that the larger *l*-BNR[2,*n*]/*c*-BNR[2,*n*] should possess increasing polyradical character in their ground states (see Tables S2 and S3 in Supplementary Information).

### Occupation numbers of active orbitals

To illustrate why *S*_vN_ increases with the molecular size, we plot the occupation numbers of active orbitals for the ground state of *l*-BNR[2,*n*]/*c*-BNR[2,*n*], obtained from spin-restricted TAO-LDA. For *l*-BNR[2,*n*]/*c*-BNR[2,*n*], the highest occupied molecular orbital, which is the (*N*/2)^th^ orbital, is referred to as the HOMO, the lowest unoccupied molecular orbital, which is the (*N*/2 + 1)^th^ orbital, is referred to as the LUMO, and so on^46,48,50–53^, where *N* is the number of electrons in *l*-BNR[2,*n*]/*c*-BNR[2,*n*].

**Figure 5:**
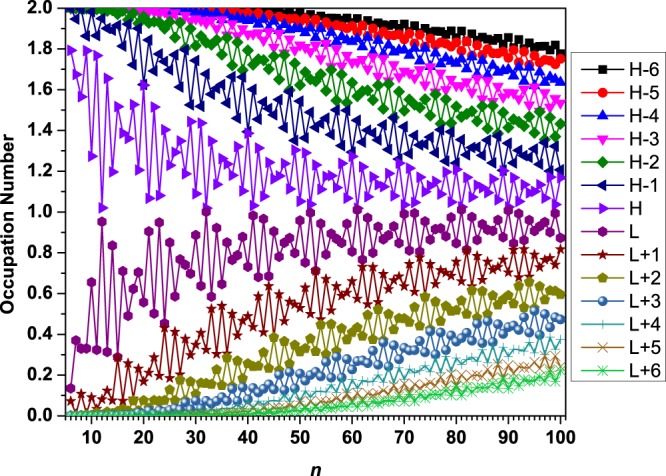
Occupation numbers of active orbitals for the ground state of *l*-BNR[2,*n*] (*n* = 6–100), obtained from spin-restricted TAO-LDA. Here, for simplicity, HOMO and LUMO are referred to as H and L, respectively.

**Figure 6:**
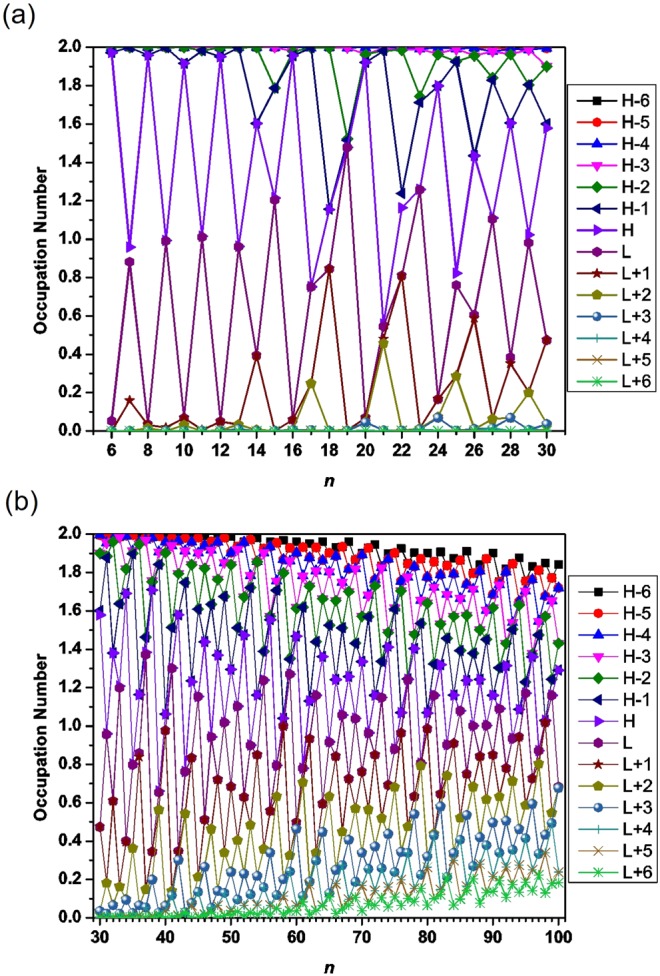
Occupation numbers of active orbitals for the ground state of *c*-BNR[2,*n*] ((**a**) *n* = 6–30 and (**b**) *n* = 30–100), obtained from spin-restricted TAO-LDA. Here, for simplicity, HOMO and LUMO are referred to as H and L, respectively.

As presented in Figs 5 and 6, the occupation numbers of active orbitals for the ground state of *c*-BNR[2,*n*] are distinctively different from those for the ground state of *l*-BNR[2,*n*]. With the increase of molecular size, there are more and more orbitals with an occupation number close to 1 (i.e., there are more and more spin-orbitals with an occupation number close to 0.5), obviously supporting that the polyradical character of the ground states of *l*-BNR[2,*n*] and *c*-BNR[2,*n*] should increase with *n*.

It is interesting to note that *c*-BNR[2,*n*] with *n* = 7, 9, 11, and 13 possess pronounced diradical character in their ground states. Besides, as the active orbital occupation numbers are close to either 0 (unoccupied) or 2 (doubly occupied), *c*-BNR[2,*n*] with *n* = 6, 8, 10, 12, 16, and 20 are expected to possess non-radical character in their ground states, showing consistency with the other electronic properties (e.g., the larger *E*_ST_ values, larger *E*_*g*_ values, and smaller *S*_vN_ values) associated with these relatively stable molecules.

### Visualization of active orbitals

**Figure 7:**
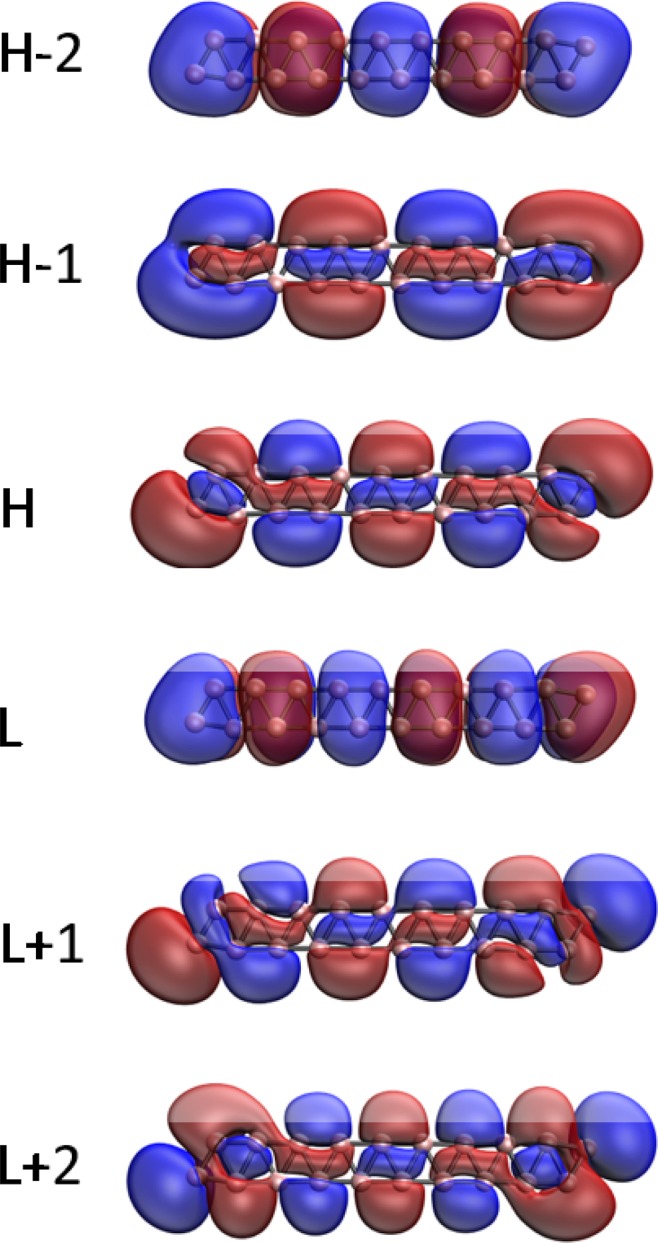
Visualization of the HOMO −2 (1.995), HOMO −1 (1.982), HOMO (1.273), LUMO (0.655), LUMO +1 (0.091), and LUMO +2 (0.003) for the ground state of *l*-BNR[2,10], obtained from spin-restricted TAO-LDA, at isovalue = 0.02 e/Å^3^. The orbital occupation numbers are shown in parentheses. Here, for simplicity, HOMO and LUMO are referred to as H and L, respectively.

**Figure 8:**
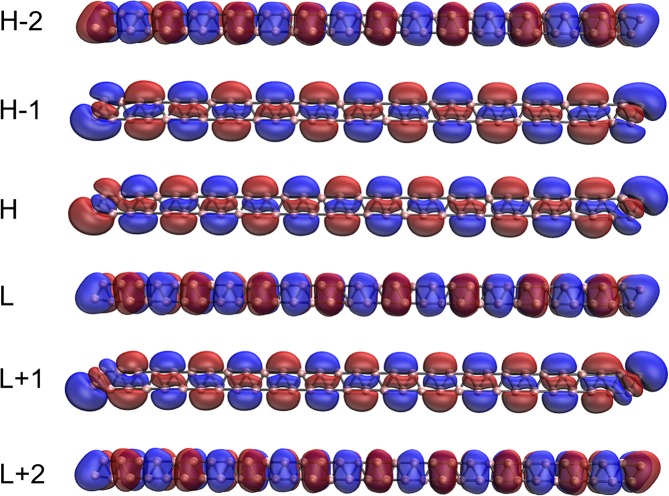
Visualization of the HOMO −2 (1.815), HOMO −1 (1.743), HOMO (1.089), LUMO (0.887), LUMO +1 (0.348), and LUMO +2 (0.106) for the ground state of *l*-BNR[2,30], obtained from spin-restricted TAO-LDA, at isovalue = 0.02 e/Å^3^. The orbital occupation numbers are shown in parentheses. Here, for simplicity, HOMO and LUMO are referred to as H and L, respectively.

**Figure 9:**
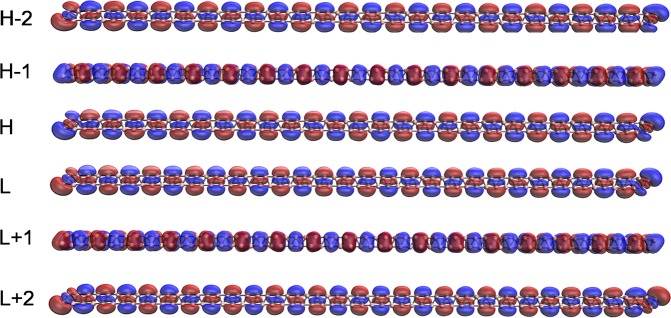
Visualization of the HOMO −2 (1.634), HOMO −1 (1.294), HOMO (1.273), LUMO (0.806), LUMO +1 (0.661), and LUMO +2 (0.409) for the ground state of *l*-BNR[2,60], obtained from spin-restricted TAO-LDA, at isovalue = 0.02 e/Å^3^. The orbital occupation numbers are shown in parentheses. Here, for simplicity, HOMO and LUMO are referred to as H and L, respectively.

**Figure 10:**
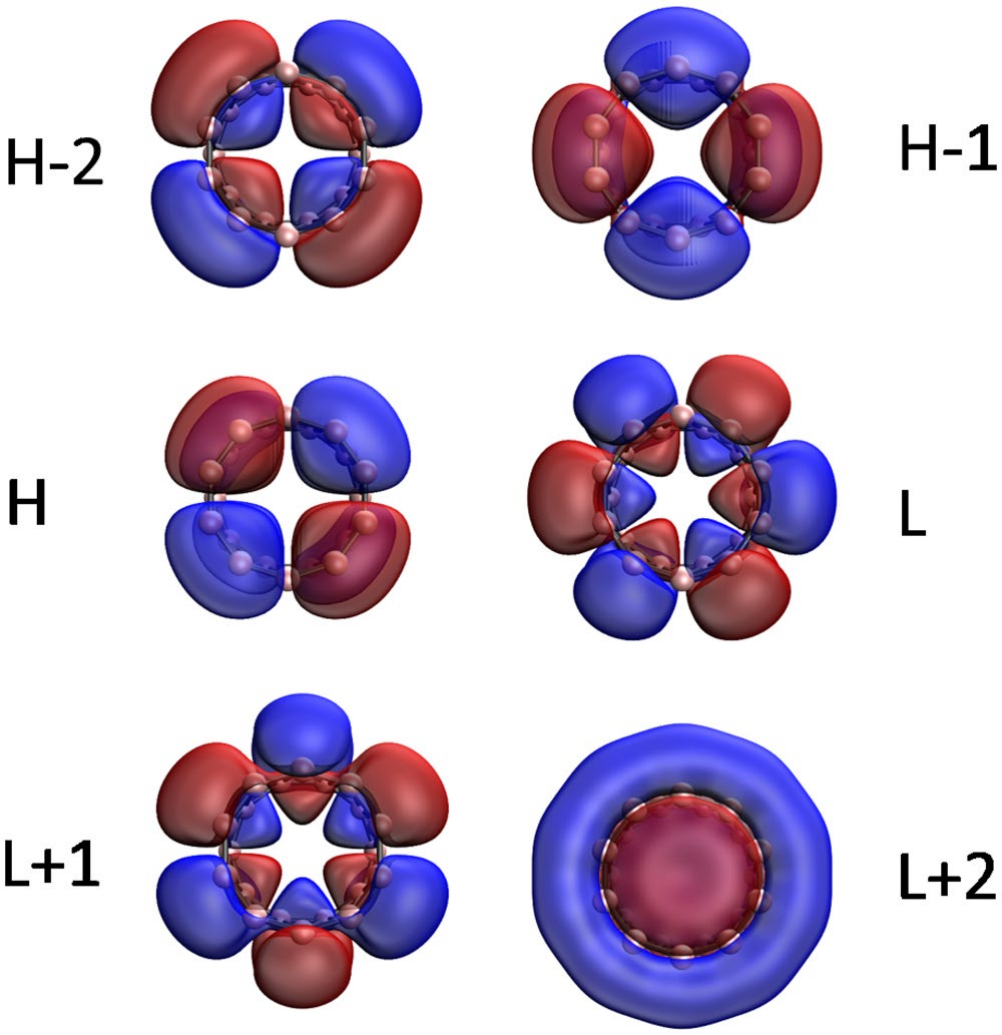
Visualization of the HOMO −2 (2.000), HOMO −1 (1.916), HOMO (1.916), LUMO (0.068), LUMO +1 (0.068), and LUMO +2 (0.030) for the ground state of *c*-BNR[2,10], obtained from spin-restricted TAO-LDA, at isovalue = 0.02 e/Å^3^. The orbital occupation numbers are shown in parentheses. Here, for simplicity, HOMO and LUMO are referred to as H and L, respectively.

**Figure 11:**
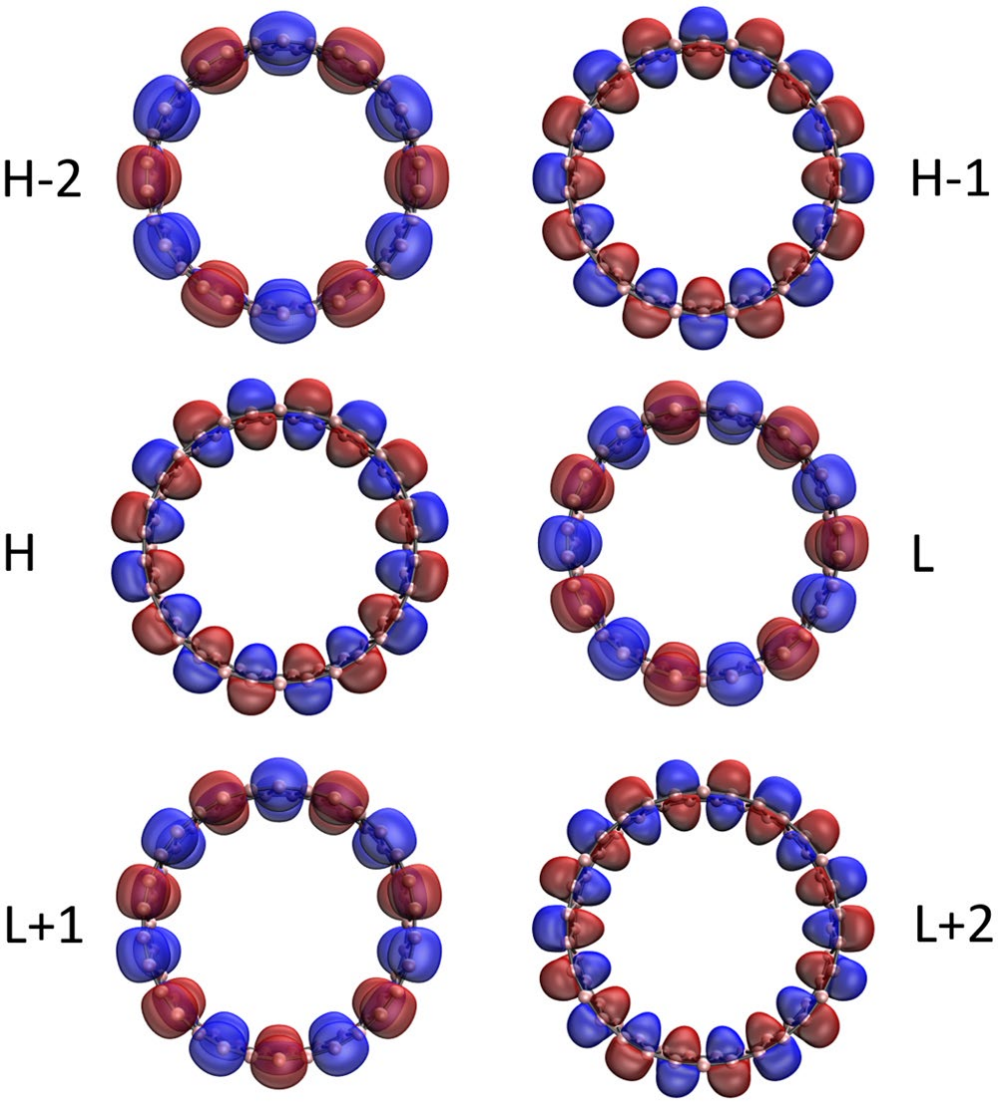
Visualization of the HOMO −2 (1.841), HOMO −1 (1.296), HOMO (1.296), LUMO (0.685), LUMO +1 (0.685), and LUMO +2 (0.240) for the ground state of *c*-BNR[2,30], obtained from spin-restricted TAO-LDA, at isovalue = 0.02 e/Å^3^. The orbital occupation numbers are shown in parentheses. Here, for simplicity, HOMO and LUMO are referred to as H and L, respectively.

**Figure 12:**
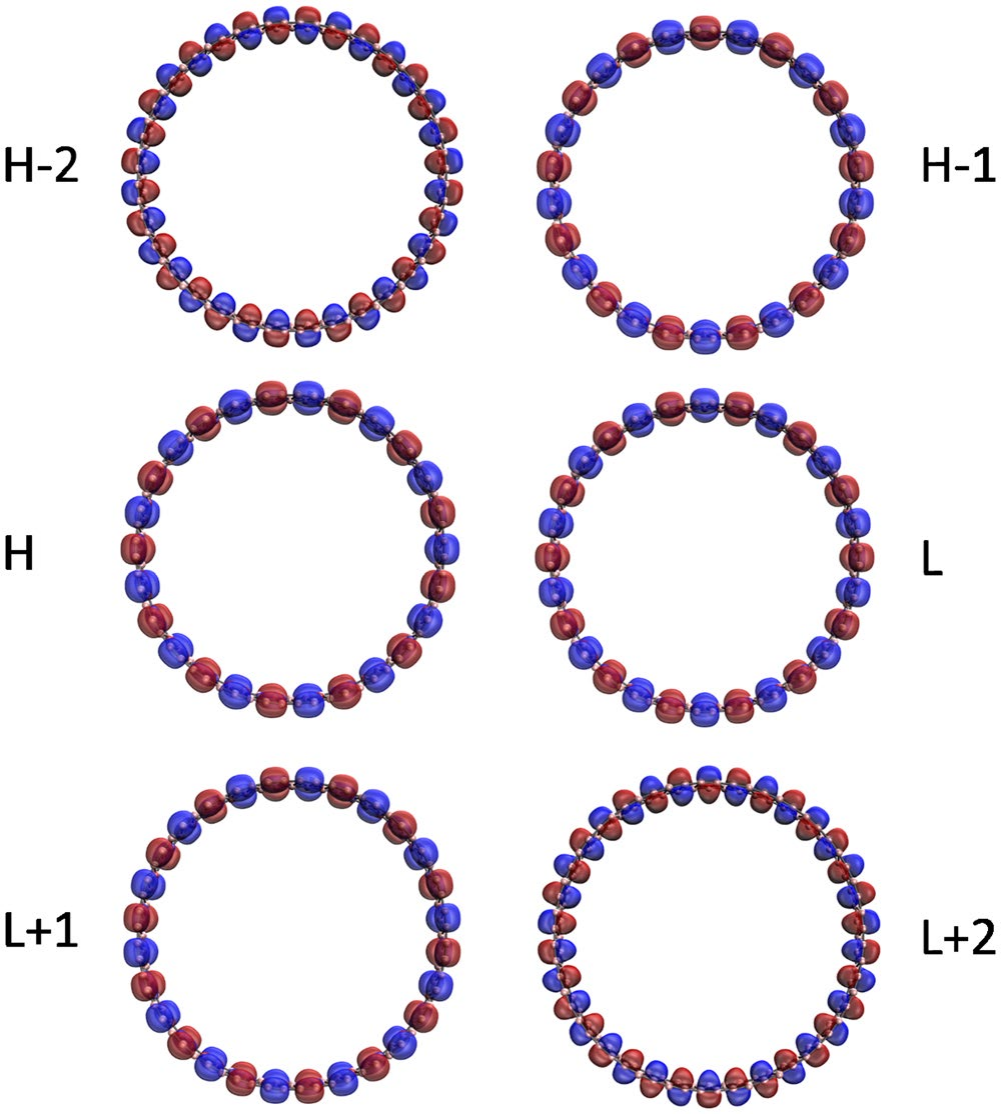
Visualization of the HOMO −2 (1.614), HOMO −1 (1.468), HOMO (1.468), LUMO (0.498), LUMO +1 (0.498), and LUMO +2 (0.464) for the ground state of *c*-BNR[2,60], obtained from spin-restricted TAO-LDA, at isovalue = 0.02 e/Å^3^. The orbital occupation numbers are shown in parentheses. Here, for simplicity, HOMO and LUMO are referred to as H and L, respectively.

Here, we investigate the visualization of the active orbitals (e.g., HOMO −2, HOMO −1, HOMO, LUMO, LUMO +1, and LUMO +2) for the ground states of a few illustrative *l*-BNR[2,*n*]/*c*-BNR[2,*n*] (e.g., *n* = 10, 30, and 60), obtained from spin-restricted TAO-LDA. As shown, the active orbitals are delocalized along the length of *l*-BNR[2,*n*] (see Figs 7–9) or the circumference of *c*-BNR[2,*n*] (see Figs 10–12).

Note that electron delocalization is a phenomenon in which electrons in a molecule are not associated with specific atoms or bonds, but are spread out over many atoms or bonds. As delocalized electrons are distributed over a greater region of space (e.g., many atoms or bonds), the net energy of the molecule is lowered, yielding resonance stabilization. Therefore, electron delocalization is an energetically favorable process^66^. Moreover, since materials with several delocalized electrons tend to be highly conductive^67^, the delocalized electrons of the boron nanoribbons are expected to enable enhanced electrical conductivity.

### Relative stability

As mentioned previously, *c*-BNR[2,*n*] possesses larger *E*_ST_ and *E*_*g*_ values than *l*-BNR[2,*n*], implying that *c*-BNR[2,*n*] should be more stable than *l*-BNR[2,*n*]. Here, we assess the relative stability of the two isomers, i.e., *l*-BNR[2,*n*] and *c*-BNR[2,*n*] by calculating the relative energy of *l*-BNR[2,*n*] with respect to *c*-BNR[2,*n*].

6 $${E}_{rel}={E}_{{\rm{S}}}({l}-{\rm{B}}{\rm{N}}{\rm{R}})-{{\rm{E}}}_{{\rm{S}}}({c}-{\rm{B}}{\rm{N}}{\rm{R}}),$$

where *E*_S_(*l*-BNR) and *E*_S_(*c*-BNR) are the ground-state (i.e., the lowest singlet state) energies of *l*-BNR[2,*n*] and *c*-BNR[2,*n*], respectively, obtained from spin-unrestricted TAO-LDA.

**Figure 13:**
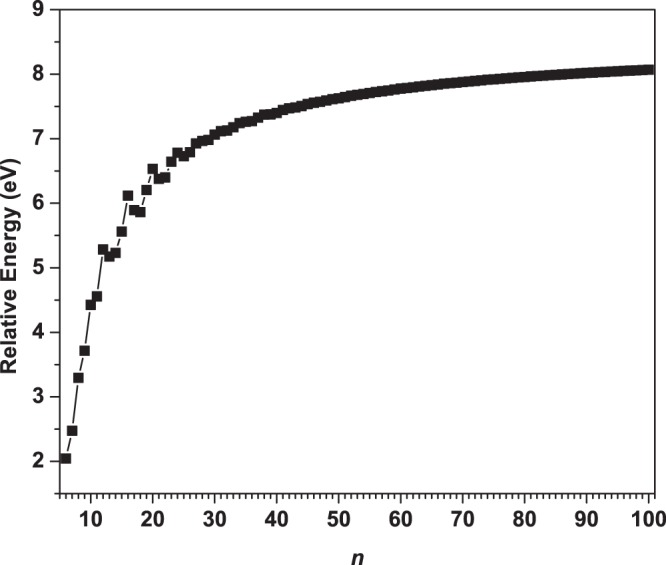
Relative energy of *l*-BNR[2,*n*] with respect to *c*-BNR[2,*n*] (*n* = 6–100), obtained from spin-unrestricted TAO-LDA.

As presented in Fig. 13, with the increase of molecular size, *E*_*rel*_ generally monotonically increases (with a slight oscillatory pattern only for the smaller *n* values (*n* ≤ 30)). Besides, *c*-BNR[2,*n*] are indeed more stable than *l*-BNR[2,*n*] for all the *n* values studied (see Table S4 in Supplementary Information), revealing the role of cyclic topology.

## Conclusions

In summary, nanosystems with radical nature, which are typically beyond the reach of traditional computational methods, have been accessible due to recent advances in TAO-DFT. In this work, we have employed TAO-DFT to predict the electronic properties (e.g., *E*_ST_, IP_*v*_, EA_*v*_, *E*_*g*_, *S*_vN_, active orbital occupation numbers, visualization of active orbitals, and relative stability) of *l*-BNR[2,*n*] and *c*-BNR[2,*n*], with *n* ranging from 6 to 100. Since the ground states of the larger *l*-BNR[2,*n*] and *c*-BNR[2,*n*] have been shown to exhibit polyradical character, calculations based on KS-DFT with conventional XC functionals may not reliably predict their electronic properties, and calculations based on accurate MR computational approaches are computationally infeasible for the larger *l*-BNR[2,*n*] and *c*-BNR[2,*n*]. Therefore, adopting TAO-DFT in the present study is well justified.

On the basis of our TAO-DFT results, *l*-BNR[2,*n*] and *c*-BNR[2,*n*] have singlet ground states for all the *n* values investigated. The electronic properties of *c*-BNR[2,*n*] exhibit more pronounced oscillatory patterns than those of *l*-BNR[2,*n*] when *n* is small, and approach the respective properties of *l*-BNR[2,*n*] when *n* is sufficiently large. It is interesting to note that *c*-BNR[2,*n*] with *n* = 6, 8, 10, 12, 16, and 20 possess non-radical character, and *c*-BNR[2,*n*] with *n* = 7, 9, 11, and 13 possess pronounced diradical character in their ground states. Besides, the larger *l*-BNR[2,*n*]/*c*-BNR[2,*n*], which have the smaller *E*_ST_ values, smaller *E*_*g*_ values, larger *S*_vN_ values, and more pronounced polyradical character, should exhibit stronger static correlation effects than the smaller *l*-BNR[2,*n*]/*c*-BNR[2,*n*]. In addition, the visualization of active orbitals has revealed that the active orbitals are delocalized along the length of *l*-BNR[2,*n*] or the circumference of *c*-BNR[2,*n*]. From the relative stability of the two isomers, *c*-BNR[2,*n*] are more stable than *l*-BNR[2,*n*] for all the *n* values studied, revealing the role of cyclic topology. Owing to their size-dependent electronic properties, *l*-BNR[2,*n*] and *c*-BNR[2,*n*] can be promising for nanoelectronics applications.

## Supplementary information

Supplementary information
