# Supplementary material for: Electronic Properties of Linear and Cyclic Boron Nanoribbons from Thermally-Assisted-Occupation Density Functional Theory
Source: Sci Rep. 2019 Aug 20;9:12139. doi: 10.1038/s41598-019-48560-z (PMC6702209; doi:10.1038/s41598-019-48560-z)
Supplement: Supplementary file 1 — Supplementary information [file 41598_2019_48560_MOESM1_ESM.pdf]

Supplementary Information to: Electronic Properties of Linear and Cyclic Boron Nanoribbons from Thermally-Assisted-Occupation Density Functional Theory

Sonai Seenithurai[1] and Jeng-Da Chai[1,2,*]

[1]*Department of Physics, National Taiwan University, Taipei 10617, Taiwan*

[2]*Center for Theoretical Physics and Center for Quantum Science and Engineering, National Taiwan University, Taipei 10617, Taiwan*

[*] Author to whom correspondence should be addressed. Electronic mail: jdchai@phys.ntu.edu.tw

LIST OF TABLES

S1 Singlet-triplet energy gap E_{ST} (in kcal/mol) of l-BNR[2,n]/c-BNR[2,n], obtained from spin-unrestricted TAO-LDA. 3

S2 Vertical ionization potential IP$_v$ (in eV), vertical electron affinity EA$_v$ (in eV), fundamental gap E_g (in eV), and symmetrized von Neumann entropy S_{vN} for the ground state of l-BNR[2,n], obtained from spin-unrestricted TAO-LDA. . 6

S3 Vertical ionization potential IP$_v$ (in eV), vertical electron affinity EA$_v$ (in eV), fundamental gap E_g (in eV), and symmetrized von Neumann entropy S_{vN} for the ground state of c-BNR[2,n], obtained from spin-unrestricted TAO-LDA. . 9

S4 Relative energy E_{rel} (in eV) of l-BNR[2,n] with respect to c-BNR[2,n], obtained from spin-unrestricted TAO-LDA. 13

TABLES

TABLE S1. Singlet-triplet energy gap E_{ST} (in kcal/mol) of l-BNR[2,n]/c-BNR[2,n], obtained from spin-unrestricted TAO-LDA.

n	E_{ST} (l-BNR[2,n])	E_{ST} (c-BNR[2,n])
6	5.83	25.33
7	5.21	5.29
8	3.83	18.57
9	5.42	7.33
10	2.95	14.83
11	4.95	7.69
12	2.83	18.80
13	4.20	7.57
14	2.94	5.46
15	3.49	6.38
16	3.02	16.50
17	2.98	6.11
18	2.97	3.57
19	2.68	5.78
20	2.82	12.03
21	2.51	5.30
22	2.63	3.75
23	2.40	5.67
24	2.43	7.79
25	2.31	4.81
26	2.27	4.29
27	2.22	5.23
28	2.15	5.15
29	2.12	4.32

30	2.04	4.57
31	2.02	4.51
32	1.96	3.88
33	1.93	3.92
34	1.88	4.38
35	1.85	3.86
36	1.81	3.33
37	1.77	3.61
38	1.74	3.90
39	1.71	3.38
40	1.68	3.09
41	1.65	3.33
42	1.62	3.38
43	1.59	3.04
44	1.57	2.94
45	1.54	3.05
46	1.52	2.97
47	1.49	2.80
48	1.47	2.78
49	1.45	2.78
50	1.43	2.66
51	1.41	2.60
52	1.39	2.61
53	1.37	2.54
54	1.35	2.45
55	1.33	2.43
56	1.31	2.42
57	1.29	2.35
58	1.28	2.29
59	1.26	2.28

60	1.24	2.25
61	1.23	2.19
62	1.21	2.15
63	1.20	2.14
64	1.19	2.10
65	1.17	2.05
66	1.16	2.03
67	1.15	2.01
68	1.13	1.97
69	1.12	1.94
70	1.11	1.92
71	1.10	1.89
72	1.09	1.86
73	1.07	1.83
74	1.06	1.81
75	1.05	1.79
76	1.04	1.76
77	1.03	1.74
78	1.02	1.72
79	1.01	1.70
80	1.00	1.67
81	0.99	1.65
82	0.98	1.63
83	0.97	1.61
84	0.96	1.59
85	0.95	1.58
86	0.95	1.56
87	0.94	1.54
88	0.93	1.52
89	0.92	1.51

90	0.91	1.49
91	0.91	1.47
92	0.90	1.46
93	0.89	1.44
94	0.88	1.42
95	0.88	1.41
96	0.87	1.40
97	0.86	1.38
98	0.86	1.37
99	0.85	1.35
100	0.84	1.34

TABLE S2. Vertical ionization potential IP_v (in eV), vertical electron affinity EA_v (in eV), fundamental gap E_g (in eV), and symmetrized von Neumann entropy $S_{\rm vN}$ for the ground state of l-BNR[2,n], obtained from spin-unrestricted TAO-LDA.

n	IP_v	EA_v	E_g	$S_{\rm vN}$
6	7.67	2.78	4.89	1.50
7	7.59	3.14	4.45	2.17
8	7.27	3.13	4.14	2.44
9	7.28	3.33	3.96	2.20
10	7.03	3.40	3.64	3.12
11	7.03	3.47	3.56	2.42
12	6.87	3.58	3.29	3.47
13	6.83	3.61	3.22	2.87
14	6.73	3.71	3.02	3.67
15	6.67	3.73	2.93	3.44
16	6.62	3.81	2.80	3.86
17	6.55	3.84	2.70	3.98
18	6.51	3.90	2.61	4.11
19	6.45	3.93	2.52	4.43

20	6.42	3.97	2.45	4.45
21	6.37	4.00	2.36	4.81
22	6.34	4.03	2.30	4.84
23	6.30	4.07	2.23	5.16
24	6.27	4.09	2.17	5.25
25	6.24	4.12	2.11	5.50
26	6.21	4.14	2.06	5.66
27	6.18	4.17	2.01	5.85
28	6.15	4.19	1.96	6.06
29	6.13	4.21	1.92	6.22
30	6.10	4.23	1.87	6.44
31	6.08	4.25	1.83	6.60
32	6.06	4.27	1.79	6.81
33	6.04	4.29	1.76	6.98
34	6.02	4.30	1.72	7.18
35	6.01	4.32	1.69	7.37
36	5.99	4.33	1.65	7.56
37	5.97	4.35	1.62	7.75
38	5.96	4.36	1.59	7.93
39	5.94	4.38	1.56	8.13
40	5.93	4.39	1.54	8.31
41	5.91	4.40	1.51	8.50
42	5.90	4.41	1.49	8.69
43	5.88	4.42	1.46	8.88
44	5.87	4.43	1.44	9.07
45	5.86	4.45	1.41	9.26
46	5.85	4.46	1.39	9.45
47	5.84	4.47	1.37	9.64
48	5.83	4.48	1.35	9.82
49	5.82	4.48	1.33	10.01

50	5.81	4.49	1.31	10.20
51	5.80	4.50	1.29	10.39
52	5.79	4.51	1.28	10.58
53	5.78	4.52	1.26	10.77
54	5.77	4.53	1.24	10.96
55	5.76	4.53	1.23	11.15
56	5.75	4.54	1.21	11.34
57	5.74	4.55	1.19	11.52
58	5.74	4.56	1.18	11.71
59	5.73	4.56	1.16	11.90
60	5.72	4.57	1.15	12.09
61	5.71	4.58	1.14	12.28
62	5.71	4.58	1.12	12.47
63	5.70	4.59	1.11	12.66
64	5.69	4.59	1.10	12.85
65	5.69	4.60	1.09	13.03
66	5.68	4.61	1.07	13.22
67	5.67	4.61	1.06	13.41
68	5.67	4.62	1.05	13.60
69	5.66	4.62	1.04	13.79
70	5.65	4.63	1.03	13.98
71	5.65	4.63	1.02	14.17
72	5.64	4.64	1.01	14.36
73	5.64	4.64	1.00	14.55
74	5.63	4.65	0.99	14.74
75	5.63	4.65	0.98	14.92
76	5.62	4.66	0.97	15.11
77	5.62	4.66	0.96	15.30
78	5.61	4.66	0.95	15.49
79	5.61	4.67	0.94	15.68

80	5.60	4.67	0.93	15.87
81	5.60	4.68	0.92	16.06
82	5.59	4.68	0.91	16.25
83	5.59	4.68	0.91	16.43
84	5.59	4.69	0.90	16.62
85	5.58	4.69	0.89	16.81
86	5.58	4.70	0.88	17.00
87	5.57	4.70	0.87	17.19
88	5.57	4.70	0.87	17.38
89	5.57	4.71	0.86	17.57
90	5.56	4.71	0.85	17.76
91	5.56	4.71	0.84	17.94
92	5.55	4.72	0.84	18.13
93	5.55	4.72	0.83	18.32
94	5.55	4.72	0.82	18.51
95	5.54	4.73	0.82	18.70
96	5.54	4.73	0.81	18.89
97	5.54	4.73	0.80	19.08
98	5.53	4.73	0.80	19.27
99	5.53	4.74	0.79	19.46
100	5.53	4.74	0.79	19.65

TABLE S3. Vertical ionization potential IP_v (in eV), vertical electron affinity EA_v (in eV), fundamental gap E_g (in eV), and symmetrized von Neumann entropy S_{vN} for the ground state of c-BNR[2,n], obtained from spin-unrestricted TAO-LDA.

n	IP_v	EA_v	E_g	S_{vN}
6	7.61	1.42	6.19	0.55
7	6.78	1.83	4.94	3.32
8	7.23	1.85	5.38	0.83
9	6.95	2.19	4.76	2.91

10	6.92	2.21	4.71	1.46
11	6.88	2.67	4.21	3.02
12	7.04	2.49	4.55	0.98
13	6.61	2.67	3.94	3.23
14	6.60	2.95	3.64	4.04
15	6.76	3.21	3.55	4.09
16	6.81	2.93	3.88	1.29
17	6.41	3.09	3.32	4.30
18	6.46	3.37	3.09	5.56
19	6.58	3.49	3.09	4.65
20	6.56	3.28	3.28	2.11
21	6.32	3.42	2.89	4.86
22	6.36	3.61	2.75	5.67
23	6.41	3.65	2.76	4.64
24	6.35	3.57	2.78	3.50
25	6.26	3.67	2.59	5.16
26	6.27	3.77	2.50	5.50
27	6.27	3.78	2.49	4.92
28	6.21	3.79	2.43	5.05
29	6.19	3.85	2.34	5.73
30	6.19	3.89	2.30	5.59
31	6.15	3.90	2.25	5.67
32	6.12	3.94	2.18	6.30
33	6.12	3.98	2.13	6.39
34	6.10	3.99	2.11	6.05
35	6.06	4.01	2.05	6.58
36	6.05	4.05	1.99	7.19
37	6.05	4.08	1.97	7.02
38	6.03	4.08	1.95	6.81
39	6.00	4.10	1.89	7.44

40	5.99	4.14	1.85	7.85
41	5.98	4.15	1.83	7.67
42	5.96	4.16	1.80	7.71
43	5.94	4.18	1.76	8.22
44	5.93	4.21	1.73	8.44
45	5.92	4.22	1.70	8.40
46	5.91	4.23	1.68	8.62
47	5.90	4.25	1.64	8.95
48	5.89	4.27	1.62	9.07
49	5.87	4.28	1.60	9.18
50	5.86	4.29	1.57	9.46
51	5.85	4.31	1.55	9.67
52	5.84	4.32	1.53	9.77
53	5.83	4.33	1.50	9.98
54	5.82	4.34	1.48	10.25
55	5.81	4.35	1.46	10.39
56	5.81	4.36	1.44	10.52
57	5.79	4.37	1.42	10.76
58	5.79	4.39	1.40	10.99
59	5.78	4.40	1.38	11.13
60	5.77	4.40	1.37	11.29
61	5.76	4.41	1.35	11.53
62	5.75	4.42	1.33	11.73
63	5.75	4.43	1.32	11.88
64	5.74	4.44	1.30	12.07
65	5.73	4.45	1.28	12.29
66	5.73	4.46	1.27	12.46
67	5.72	4.47	1.25	12.64
68	5.71	4.47	1.24	12.84
69	5.71	4.48	1.22	13.04

70	5.70	4.49	1.21	13.21
71	5.69	4.50	1.20	13.40
72	5.69	4.50	1.18	13.60
73	5.68	4.51	1.17	13.78
74	5.68	4.52	1.16	13.96
75	5.67	4.52	1.15	14.16
76	5.66	4.53	1.14	14.35
77	5.66	4.53	1.12	14.54
78	5.65	4.54	1.11	14.72
79	5.65	4.55	1.10	14.92
80	5.64	4.55	1.09	15.11
81	5.64	4.56	1.08	15.29
82	5.63	4.56	1.07	15.48
83	5.63	4.57	1.06	15.67
84	5.62	4.57	1.05	15.86
85	5.62	4.58	1.04	16.05
86	5.62	4.58	1.03	16.24
87	5.61	4.59	1.02	16.43
88	5.61	4.59	1.01	16.61
89	5.60	4.60	1.00	16.80
90	5.60	4.60	0.99	16.99
91	5.59	4.61	0.98	17.18
92	5.59	4.61	0.98	17.37
93	5.59	4.62	0.97	17.56
94	5.58	4.62	0.96	17.75
95	5.58	4.63	0.95	17.94
96	5.57	4.63	0.94	18.13
97	5.57	4.63	0.94	18.31
98	5.57	4.64	0.93	18.50
99	5.56	4.64	0.92	18.69

100	5.56	4.65	0.91	18.88

TABLE S4. Relative energy E_{rel} (in eV) of l-BNR[2,n] with respect to c-BNR[2,n], obtained from spin-unrestricted TAO-LDA.

n	E_{rel}
6	2.04
7	2.47
8	3.29
9	3.72
10	4.42
11	4.56
12	5.28
13	5.18
14	5.23
15	5.56
16	6.12
17	5.89
18	5.86
19	6.21
20	6.53
21	6.38
22	6.40
23	6.64
24	6.78
25	6.73
26	6.79
27	6.93
28	6.97
29	6.98
30	7.06

31	7.12
32	7.13
33	7.18
34	7.24
35	7.26
36	7.27
37	7.33
38	7.37
39	7.38
40	7.40
41	7.44
42	7.47
43	7.48
44	7.51
45	7.54
46	7.56
47	7.57
48	7.59
49	7.61
50	7.63
51	7.65
52	7.67
53	7.68
54	7.69
55	7.71
56	7.72
57	7.74
58	7.75
59	7.76
60	7.78

61	7.79
62	7.80
63	7.81
64	7.82
65	7.83
66	7.84
67	7.85
68	7.86
69	7.87
70	7.88
71	7.89
72	7.90
73	7.90
74	7.91
75	7.92
76	7.93
77	7.94
78	7.94
79	7.95
80	7.96
81	7.96
82	7.97
83	7.98
84	7.98
85	7.99
86	8.00
87	8.00
88	8.01
89	8.01
90	8.02

91	8.03
92	8.03
93	8.04
94	8.04
95	8.05
96	8.05
97	8.06
98	8.06
99	8.06
100	8.07
